# A 72‐year‐old with eyelid opening apraxia in Steele–Richardson–Olszewski syndrome

**DOI:** 10.1002/ccr3.958

**Published:** 2017-04-26

**Authors:** Ihtesham A. Qureshi, Mohtashim A. Qureshi, Alberto Maud

**Affiliations:** ^1^ Neurology DepartmentTexas Tech University Health Sciences Center4800 Alberta AvenueEl Paso79905Texas

**Keywords:** Apraxia, blepharospasm, eyelid opening, progressive supranuclear palsy

## Abstract

In our patient with Steele–Richardson–Olszewski syndrome presenting with apraxia of eyelid opening, there is a possibility to be readily confused with conditions presenting in similar pattern such as blepharospasm and eyelid ptosis. Therefore, it is essential to correlate the clinical findings with electromyographic recordings and dopamine transporter scan (DaT).

A 72‐year‐old male presented with possible progressive supranuclear palsy (PSP) (or Steele–Richardson–Olszewski syndrome) based on National Institute of Neurological Disorders and Stroke Criteria for progressive supranuclear palsy (NINDS‐SPSP) exhibiting severe apraxia of eyelid opening (ALO) (Video S1) 1. The presence of ALO is under identified among patients with PSP. The pathophysiology behind ALO involves sustained contraction of pretarsal portion of the orbicularis oculi. There is a high possibility for it to be confused with blepharospasm (due to its frequent association) and ptosis of eyelid 2. Easy way to differentiate ALO from blepharospasm is, in patients with ALO, it is difficult to open the eyes at will after its closure, while patients with blepharospasm can open eyes easily. Correlating the clinical findings with synchronous levator palpebrae and orbicularis oculi electromyographic and dopamine transporter scan (DaT) recordings is helpful in establishing the diagnosis (Figs 1 and 2).

**Figure 1:**
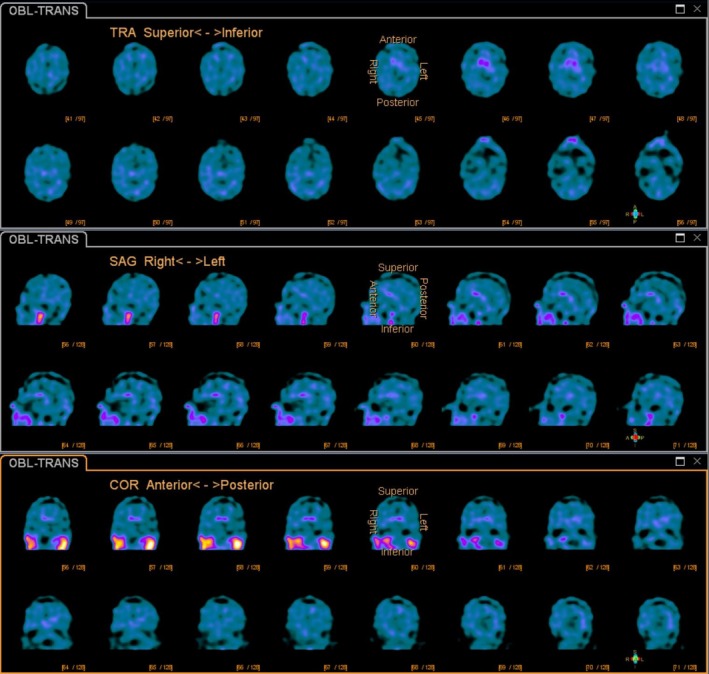
Dopamine transporter (DaT) scan SPECT. Top: Axial, Middle: Sagittal and Bottom: Coronal reformatted images show severely decreased uptake in the caudate nuclei and absent uptake in the putamina confirm depletion of dopamine supporting the diagnosis of parkinsonism.

**Figure 2:**
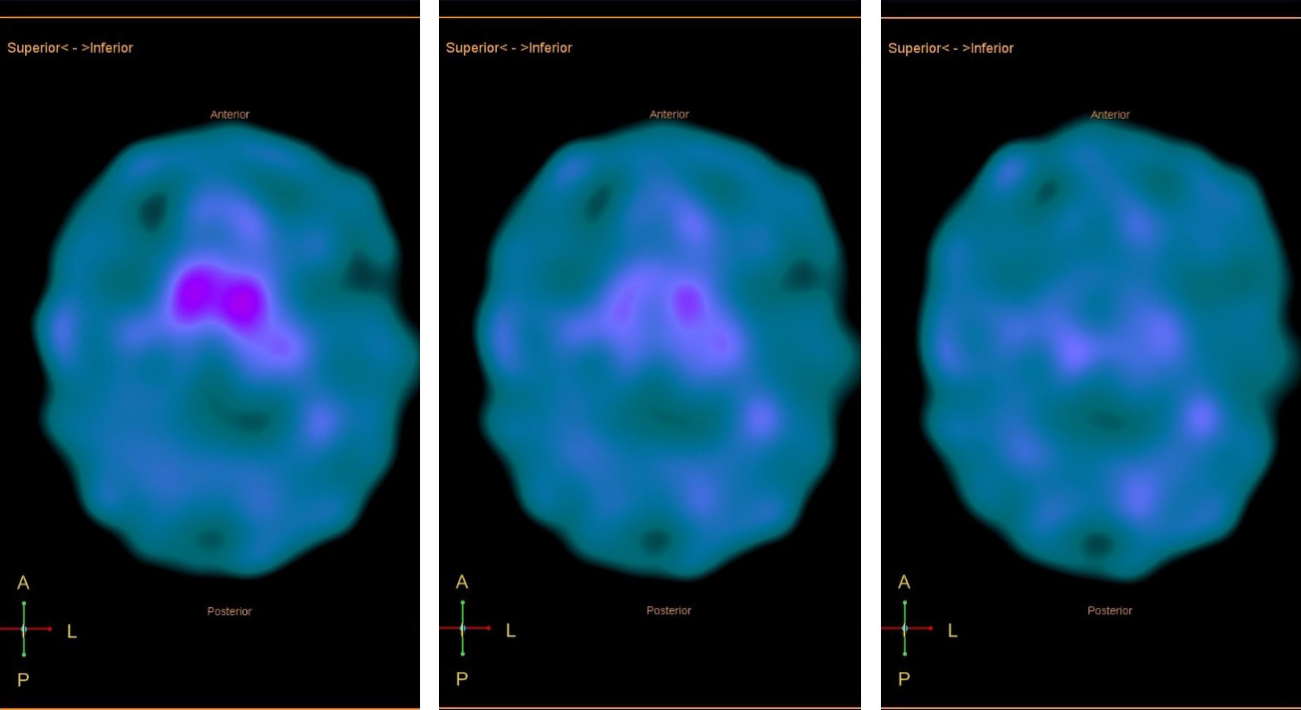
Dopamine transporter (DaT) scan SPECT images through the basal ganglia demonstrate severely decreased uptake in the caudate nuclei and absent uptake in the putamina.

## Conflict of Interest

None declared.

## Authorship

IAQ: involved in manuscript writing; MAQ: involved in critical revision of the manuscript; AM: involved in patient care.

## Supporting information


**Video S1.** The video presents a patient with Steele–Richardson–Olszewski syndrome exhibiting severe apraxia of eyelid opening.
